# Machine learning in risk prediction of continuous renal replacement therapy after coronary artery bypass grafting surgery in patients

**DOI:** 10.1007/s10157-024-02472-z

**Published:** 2024-03-27

**Authors:** Qian Zhang, Peng Zheng, Zhou Hong, Luo Li, Nannan Liu, Zhiping Bian, Xiangjian Chen, Hengfang Wu, Sheng Zhao

**Affiliations:** 1 Department of Cardiology, The First Affiliated Hospital of Nanjing Medical University, Nanjing, Jiangsu China; 2 Department of Neurology, The Second Affiliated Hospital of Jianghan University (Wuhan City Fifth Hospital), Wuhan, Hubei China; 3 Department of Cardiovascular Surgery of the First Affiliated Hospital & Institute for Cardiovascular Science, Soochow University, Suzhou, Jiangsu China; 4 Suzhou Medical College, Soochow University, Suzhou, Jiangsu China; 5 Department of Cardiovascular Surgery, The First Affiliated Hospital of Nanjing Medical University, Nanjing, Jiangsu China

**Keywords:** Machine learning, Risk factors, Continuous renal replacement therapy, Coronary artery bypass grafting, Prediction

## Abstract

**Objectives:**

This study aimed to develop machine learning models for risk prediction of continuous renal replacement therapy (CRRT) following coronary artery bypass grafting (CABG) surgery in intensive care unit (ICU) patients.

**Methods:**

We extracted CABG patients from the electronic medical record system of the hospital. The endpoint of this study was the requirement for CRRT after CABG surgery. The Boruta method was used for feature selection. Seven machine learning algorithms were developed to train models and validated using 10 fold cross-validation (CV). Model discrimination and calibration were estimated using the area under the receiver operating characteristic curve (AUC) and calibration plot, respectively. We used the SHapley Additive exPlanations (SHAP) method to illustrate the effects of the features attributed to the model and analyze the effects of individual features on the output of the mode.

**Results:**

In this study, 72 (37.89%) patients underwent CRRT, with a higher mortality compared to those patients without CRRT. The Gaussian Naïve Bayes (GNB) model with the highest AUC were considered as the final predictive model and performed best in predicting postoperative CRRT. The analysis of importance revealed that cardiac troponin T, creatine kinase isoenzyme, albumin, low-density lipoprotein cholesterol, NYHA, serum creatinine, and age were the top seven features of the GNB model. The SHAP force analysis illustrated how created model visualized individualized prediction of CRRT.

**Conclusions:**

Machine learning models were developed to predict CRRT. This contributes to the identification of risk variables for CRRT following CABG surgery in ICU patients and enables the optimization of perioperative managements for patients.

## Introduction

Coronary artery bypass grafting (CABG) as an effective way of myocardial revascularization is a remarkably successful operation and is commonly employed for patients with high-grade or complex coronary artery stenosis. However, the high incidence of postoperative complications may significantly impact both the overall quality of surgical healthcare and patient’s prognosis, as well as mortality [1]. The reported incidence of acute kidney injury (AKI) after CABG surgery ranges from approximate 30–50% [2, 3], while mild to moderate AKI frequently occurs. 2–4% of serious AKI patients are required for continuous renal replacement therapy (CRRT) after CABG surgery in intensive care unit (ICU) [4, 5]. Despite the advancements in intensive care quality and renal replacement therapy technique, short-term mortality of patients receiving CRRT remains high level, ranging from 40% to over 70% [6–8]. Therefore, identifying risk factors of postoperative CRRT in CABG patients is critical for reducing death risk.

The application of machine learning model based on artificial intelligence (AI) algorithms has gradually gained momentum in clinical practices owing to its demonstrated superior predictive performance compared to traditional analytical models [9]. This study was conducted to develop machine learning models for predicting risk factors of CRRT after CABG surgery in ICU patients, with an aim to safeguarding postoperative renal function and improving clinical outcomes.

## Methods

### Data source and patient population

A retrospective review was conducted on 190 adult patients with coronary heart disease who underwent isolated CABG surgery under the support of cardiopulmonary bypass at department of cardiovascular surgery of The First Affiliated Hospital of Nanjing Medical University from January 2013 to June 2020. The exclusion criteria were as follows: (1) patients age < 18 years; (2) patients who received CRRT prior to CABG surgery in the ICU; and (3) patients with missing information exceeding 30%. The endpoint of this study was the requirement for CRRT after CABG surgery. Patients’ data were partitioned randomly into a training set (90%) for model development and a validation set (10%) for model validation. This study complies with the Declaration of Helsinki (revised in 2013) and was approved and supervised by the Ethics Review Committee of The First Affiliated Hospital of Nanjing Medical University (2019-SR-313.A1). The informed consent of patients was waived by the Ethics Review Committee of the hospital.

### Criteria of CRRT

The criteria for initiation of CRRT were as follows: (1) more than 6 h of continuous anuria; (2) urine volume less than 200 mL for over 10 h; (3) serum potassium concentration > 6.5 mmol/L (hyperkalemia); (4) severe metabolic acidosis (pH < 7.20 despite normal or low partial pressure of carbon dioxide in arterial blood); (5) serum creatinine (sCr) ≥ 300 µmol/L; (6) volume overload (especially pulmonary edema unresponsive to diuretics); (7) clinical complications of uremia (e.g., encephalopathy, pericarditis, and neuropathy).

### Study variables and data collection

Baseline characteristics and co-morbidities of patients, such as age, gender, body mass index (BMI), smoking history, drinking history, acute myocardial infarction (AMI) history, hypertension, diabetes, chronic renal disease, atrioventricular block, atrial fibrillation (AF), and New York Heart Association (NYHA) classification, were documented. The electrocardiogram and echocardiogram records were gathered. Laboratory examinations, such as serum lipids, blood urea nitrogen (BUN), serum creatinine (sCr), myocardial injury biomarkers, albumin (ALB), and blood glucose levels, were tested upon admission. The timing of intra-aortic balloon pump (IABP) implantation, surgical duration, cardiopulmonary bypass time, length of hospital stay (LOS), length of ICU stay, statins use, and in-hospital mortality were recorded. We applied one standard transformation to collected variables: handling missing values by method of filling.

### Statistical analysis and development of machine learning models

Statistical analyses were conducted using SPSS software (version 23.0). Continuous variables are presented as mean ± standard deviation or as median (interquartile spacing), while categorical variables are presented as number (proportions). Then, Student’s t-test or the Mann–Whitney test was employed to compare the difference in continuous variables between two groups, and the Chi-square test was used to compare the difference in categorical variables between two groups. All *p* values were two-sided, with less than 0.05 indicating statistical significance.

The Boruta method was used to select critical features, and machine learning algorithms were then employed to construct training models using 10 fold cross-validation (CV), which effectively avoided overfitting and facilitated determination of optimal hyperparameters. This study included seven machine learning models: AdaBoost, LightGBM, Gaussian Naïve Bayes (GNB), Complement Naïve Bayes (CNB), multi-layer perceptron neural network (MLP), k-nearest neighbors (KNN), and support vector machine (SVM). Performance of the models was evaluated based on relevant indicators, including the area under the receiver operating characteristic curve (AUC), accuracy (ACC), sensitivity, and specificity. In general, the model with the highest AUC exhibited the best predictive capacity and was selected as the final prediction model. A calibration plot was generated to evaluate the correlation between predicted and actual clinical outcomes.

**Fig. 1:**
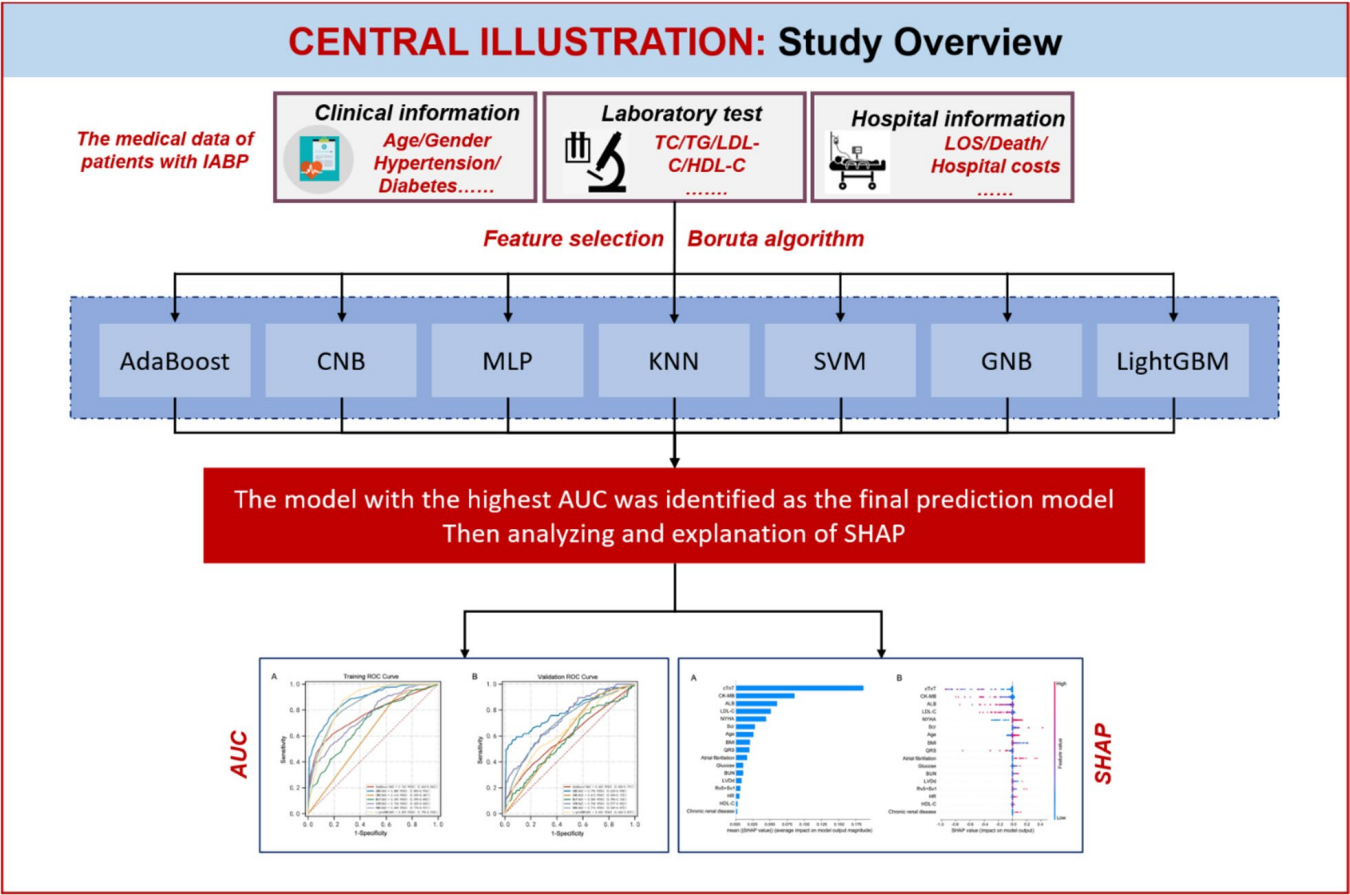
Flowchart of this study. AUC, area under the receiver operating characteristic curve; SHAP, SHapley Additive exPlanations

Furthermore, the SHapley Additive exPlanations (SHAP) method was applied to enhance the interpretability of the final model. The SHAP summary plot was used to illustrate the influence of model features. Then, the SHAP dependence plot was used to analyze the importance of individual features affecting model output. The SHAP force plot was utilized to visually represent the impact of key features on the final model in individual patients. Figure 1 illustrates the flowchart for the study.

## Results

### Baseline characteristics of patients

The baseline characteristics of the patients are shown in Table 1. This study population had 153 (80.526%) males, with their median age of 66.0 (60.0, 72.0) years old. A total of 72 patients were included in the CRRT group for this study, with a higher median age (69.0 vs 64.0 years old, *p* = *0.002*), a higher mortality (37.500% vs 7.627%, *p* < *0.001*), and a longer length of ICU (14.0 vs 11.0 d, *p* < *0.001*) compared to the non-CRRT group. The comparison of gender, BMI, hypertension, chronic renal disease, AF, NYHA classification, ALB, blood glucose, cardiac troponin T (cTnT), sCr, BUN, creatine kinase isoenzyme (CK-MB), and hospital costs detected statistical differences between the two groups (*p* ≤ *0.05*). Furthermore, there were no differences between the two groups regarding surgical duration (238 in non-CRRT group vs. 230 min in CRRT group, *p* = 0.737) and cardiopulmonary bypass time (89 in non-CRRT group vs. 94 min in CRRT group, *p* = 0.881). The detail content is shown in Table 1.

**Table 1:** Baseline characteristics of patients in the CRRT group and the non-CRRT group

		Total (*N* = 190)	Non-CRRT group (*N* = 118)	CRRT group (*N* = 72)	Statistical	P-value
Age (year)		66.0 (60.0, 72.0)	64.0 (57.0, 70.0)	69.0 (64.0, 74.0)	-3.027	**0.002**
Gender (%)	Male	153 (80.526)	89 (75.424)	64 (88.889)	5.17	**0.023**
	Female	37 (19.474)	29 (24.576)	8 (11.111)		
Smoking history (%)	No	103 (54.211)	64 (54.237)	39 (54.167)	0	0.992
	Yes	87 (45.789)	54 (45.763)	33 (45.833)		
Drinking history (%)	No	133 (70.000)	88 (74.576)	45 (62.500)	3.105	0.078
	Yes	57 (30.000)	30 (25.424)	27 (37.500)		
BMI (kg/m^2^)		23.87 (22.03, 26.01)	24.22 (23.24, 25.95)	22.49 (21.84, 26.06)	2.714	**0.007**
Number of lesions (%)	1	6 (3.158)	2 (1.695)	4 (5.556)	6.963	0.073
	2	9 (4.737)	7 (5.932)	2 (2.778)		
	3	170 (89.474)	108 (91.525)	62 (86.111)		
	4	5 (2.632)	1 (0.847)	4 (5.556)		
AMI history (%)	No	70 (36.842)	47 (39.831)	23 (31.944)	1.195	0.274
	Yes	120 (63.158)	71 (60.169)	49 (68.056)		
NYHA classification (%)	I	19 (10.000)	17 (14.407)	2 (2.778)	17.542	** < 0.001**
	II	74 (38.947)	54 (45.763)	20 (27.778)		
	III	90 (47.368)	44 (37.288)	46 (63.889)		
	VI	7 (3.684)	3 (2.542)	4 (5.556)		
Hypertension (%)	No	65 (34.211)	47 (39.831)	18 (25.000)	4.37	**0.037**
	Yes	125 (65.789)	71 (60.169)	54 (75.000)		
Diabetes (%)	No	110 (57.895)	68 (57.627)	42 (58.333)	0.009	0.924
	Yes	80 (42.105)	50 (42.373)	30 (41.667)		
Chronic renal disease (%)	No	182 (95.789)	117 (99.153)	65 (90.278)	8.732	**0.003**
	Yes	8 (4.211)	1 (0.847)	7 (9.722)		
AF (%)	No	156 (82.105)	108 (91.525)	48 (66.667)	18.807	** < 0.001**
	Yes	34 (17.895)	10 (8.475)	24 (33.333)		
Atrioventricular block (%)	No	174 (91.579)	108 (91.525)	66 (91.667)	0.001	0.973
	Yes	16 (8.421)	10 (8.475)	6 (8.333)		
Abnormal Q wave (%)	No	143 (75.263)	80 (67.797)	63 (87.500)	9.324	**0.002**
	Yes	47 (24.737)	38 (32.203)	9 (12.500)		
Abnormal ST-T segment (%)	No	28 (14.737)	20 (16.949)	8 (11.111)	1.213	0.271
	Yes	162 (85.263)	98 (83.051)	64 (88.889)		
Premature contraction (%)	No	150 (78.947)	94 (79.661)	56 (77.778)	0.095	0.757
	Yes	40 (21.053)	24 (20.339)	16 (22.222)		
Operative branch block (%)	No	170 (89.474)	106 (89.831)	64 (88.889)	0.042	0.837
	Yes	20 (10.526)	12 (10.169)	8 (11.111)		
Prolonged QT interval (%)	No	175 (92.105)	110 (93.220)	65 (90.278)	0.532	0.466
	Yes	15 (7.895)	8 (6.780)	7 (9.722)		
RV5 + SV1 (mv)		2.11 (1.75, 2.75)	2.07 (1.75, 2.65)	2.19 (1.95, 2.97)	-1.984	**0.047**
QRS interval (ms)		97.0 (92.0, 106.1)	97.0 (94.0, 107.0)	97.8 (92.0, 104.0)	1.345	0.179
HR (bmp)		74.0 (63.0, 85.0)	73.0 (64.0, 81.0)	76.0 (62.0, 90.0)	-1.206	0.228
LVDd (mm^3^)		52.0 (47.0, 56.0)	53.0 (48.0, 56.0)	50.0 (47.0, 55.0)	1.175	0.240
LVDs (mm^3^)		37.0 (32.0, 43.0)	38.5 (32.0, 43.0)	35.0 (31.0, 47.0)	0.737	0.461
LVFS (%)		29.3 (22.6, 33.3)	28.0 (22.6, 34.0)	30.4 (22.6, 33.3)	0.226	0.822
LVEF (%)		55.4 (44.6, 62.4)	53.6 (45.2, 62.4)	57.9 (44.6, 62.4)	0.174	0.863
cTnT (ng/mL)		93.8 (27.8, 369.3)	172.2 (45.1, 927.9)	49.8 (22.2, 158.1)	3.875	** < 0.001**
CK-MB (ng/mL)		11.0 (3.0, 16.0)	14.0 (8.0, 19.1)	4.6 (1.9, 11.1)	5.886	** < 0.001**
BNP (pg/mL)		1431.0 (728.9, 4067.2)	1501.5 (612.8, 3035.2)	1380.0 (892.3, 7319.0)	-1.033	0.302
BUN (mmol/L)		6.75 (5.40, 8.96)	6.02 (5.04, 7.82)	7.71 (6.44, 9.50)	-4.462	** < 0.001**
sCr (μmol/L)		77.8 (65.5, 105.4)	71.4 (62.9, 89.2)	105.7 (76.8, 134.8)	-6.116	** < 0.001**
Blood glucose (mmol/L)		5.90 (4.95, 9.46)	5.80(5.08, 7.46)	8.64(4.81, 10.73)	-2.370	**0.018**
ALB (g/L)		35.78 ± 3.83	36.30 ± 4.03	34.93 ± 3.33	2.415	**0.017**
LDL-C (mmol/L)		2.33 (1.79, 3.01)	2.38 (1.94, 3.01)	2.11 (1.50, 2.99)	2.543	**0.011**
HDL-C (mmol/L)		0.92 (0.81, 1.04)	0.91 (0.81, 1.04)	0.95 (0.81, 1.07)	-0.858	0.391
TG (mmol/L)		1.28 (0.94, 1.71)	1.30 (0.97, 1.91)	1.23 (0.94, 1.42)	2.003	**0.045**
TC (mmol/L)		3.77 (3.03, 4.66)	3.85 (3.16, 4.56)	3.51 (2.82, 4.94)	1.672	0.095
Statins (%)	No	48 (25.263)	26 (22.034)	22 (30.556)	1.72	0.190
	Yes	142 (74.737)	92 (77.966)	50 (69.444)		
Surgical duration (min)		235 (198, 282)	238 (198, 280)	230 (203, 283)	-0.337	0.737
Cardiopulmonary bypass time (min)		91 (73, 113)	89 (73, 113)	94 (72, 111)	0.151	0.881
Preoperative IABP (%)	No	112 (58.947)	70 (59.322)	42 (58.333)	0.018	0.893
	Yes	78 (41.053)	48 (40.678)	30 (41.667)		
LOS (d)		25.0 (18.0, 39.0)	26.0 (18.0, 32.0)	22.0 (18.0, 46.0)	-0.325	0.746
Length of ICU stay (d)		12.0 (8.0, 20.0)	11.0 (7.0, 19.0)	14.0 (8.0, 29.0)	-2.385	**0.017**
Hospital costs (RMB)		331,380 (238,438, 423,720)	278,449 (217,998, 381,234)	422,823 (336,081, 479,819)	-6.94	** < 0.001**
Death (%)	No	154 (81.053)	109 (92.373)	45 (62.500)	25.984	** < 0.001**
	Yes	36 (18.947)	9 (7.627)	27 (37.500)		

Bold values indicate *p*-values are statistically different

Continuous variables are presented as mean ± standard deviation or as median (interquartile spacing), while categorical variables are presented as number (proportions). Student’s t-test or the Mann–Whitney test was employed to compare the difference in continuous variables between two groups, and the Chi-square test was used to compare the difference in categorical variables between the two groups

AMI, acute myocardial infarction; NYHA, New York Heart Association; AF, atrial fibrillation; HR, heart rate; LVDs, left ventricular end-systolic dimension; LVDd, left ventricular end-diastolic dimension; LVEF, left ventricular ejection fraction; LVFS, left ventricular fraction shortening; LDL-C, low-density lipoprotein cholesterol; HDL-C, high-density lipoprotein cholesterol; TG, triglyceride; TC, total cholesterol; cTnT, cardiac troponin T; CK-MB, creatine kinase isoenzyme; BNP, B-type natriuretic peptide; BUN, blood urea nitrogen; Scr, serum creatinine; ALB, albumin; ICU, intensive care unit; IABP, intra-aortic balloon pump; LOS, length of hospital stay; Non-CRRT, patients without continuous renal replacement therapy; CRRT group, patients with continuous renal replacement therapy

### Developed machine learning models and their prediction performance

**Fig. 2:**
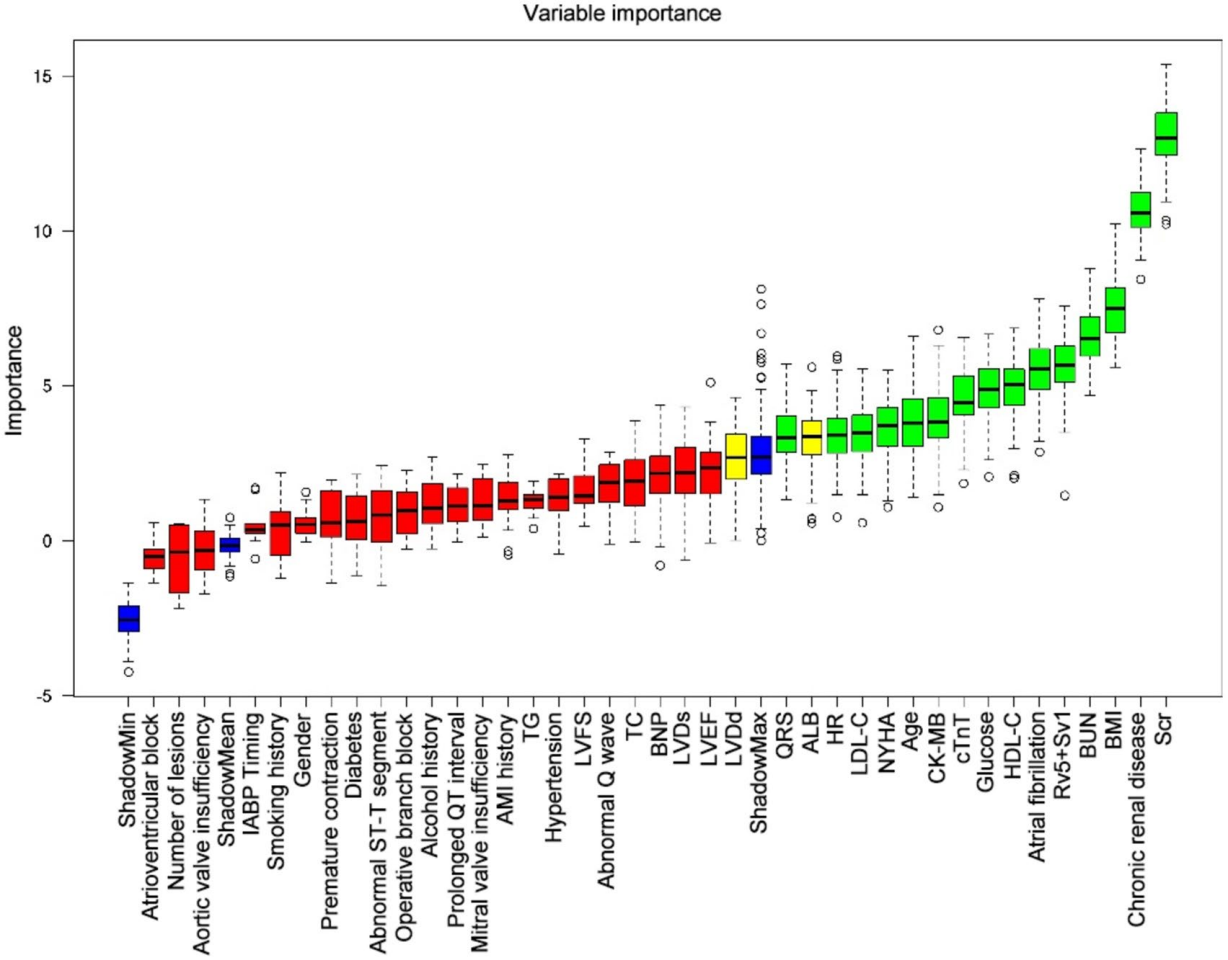
Feature selection based on the Boruta algorithm. The horizontal axis is the name of variable, and the vertical axis is the *Z*-value of each variable. The box plot exhibits the *Z*-value of each variable during model calculation. The green boxes represent the first 15 important variables, the yellow represents tentative attributes, and the red represents unimportant variables

**Fig. 3:**
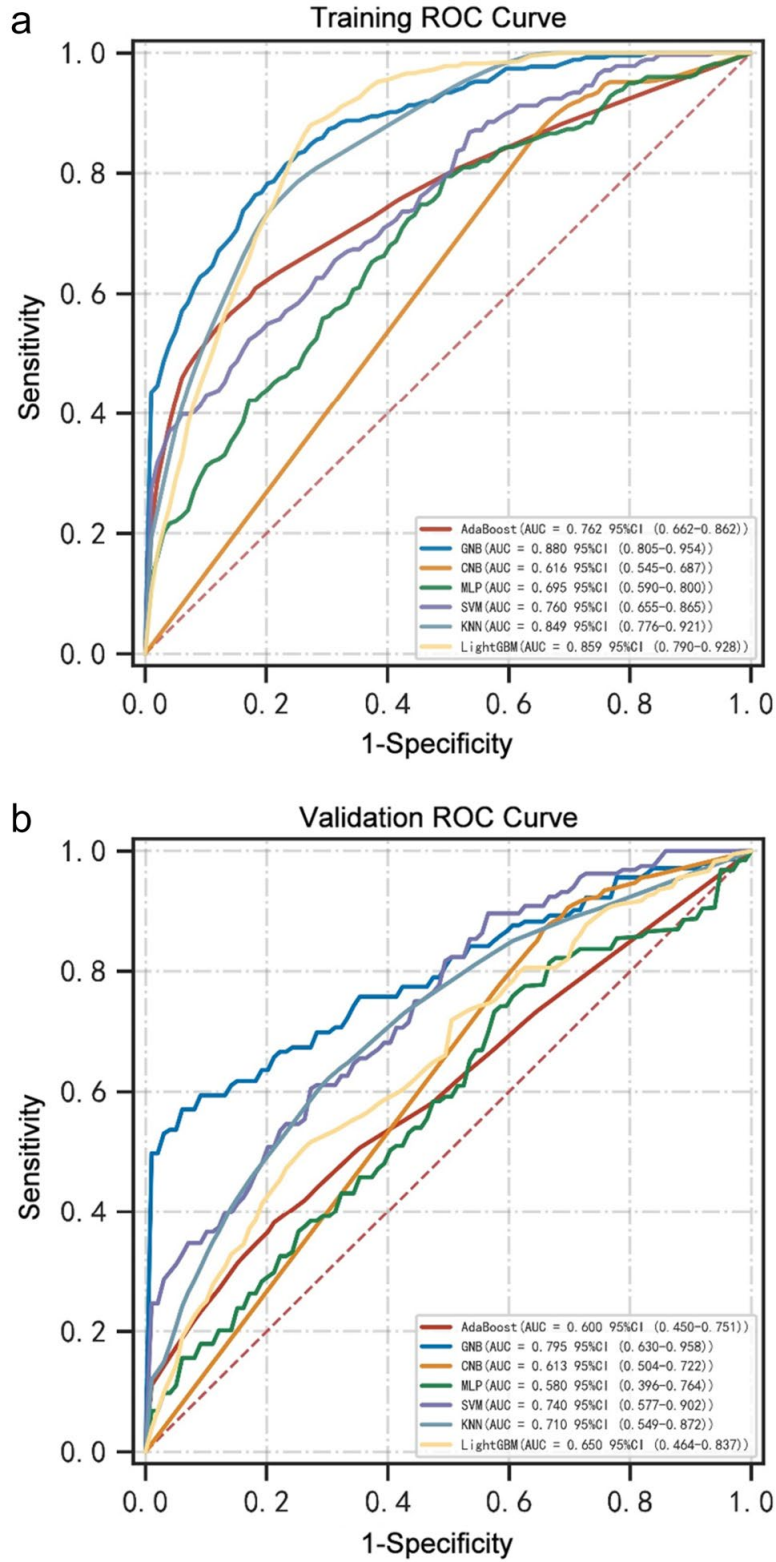
Comparison of receiver operator characteristic curves (ROCs) for the machine learning models. **a** The ROCs of training models. **b** The ROCs of validation models. AUC, area under the ROC; GNB, Gaussian Naïve Bayes; CNB, Complement Naïve Bayes; MLP, multi-layer perceptron neural network; SVM, support vector machine; KNN, k-nearest neighbors

**Fig. 4:**
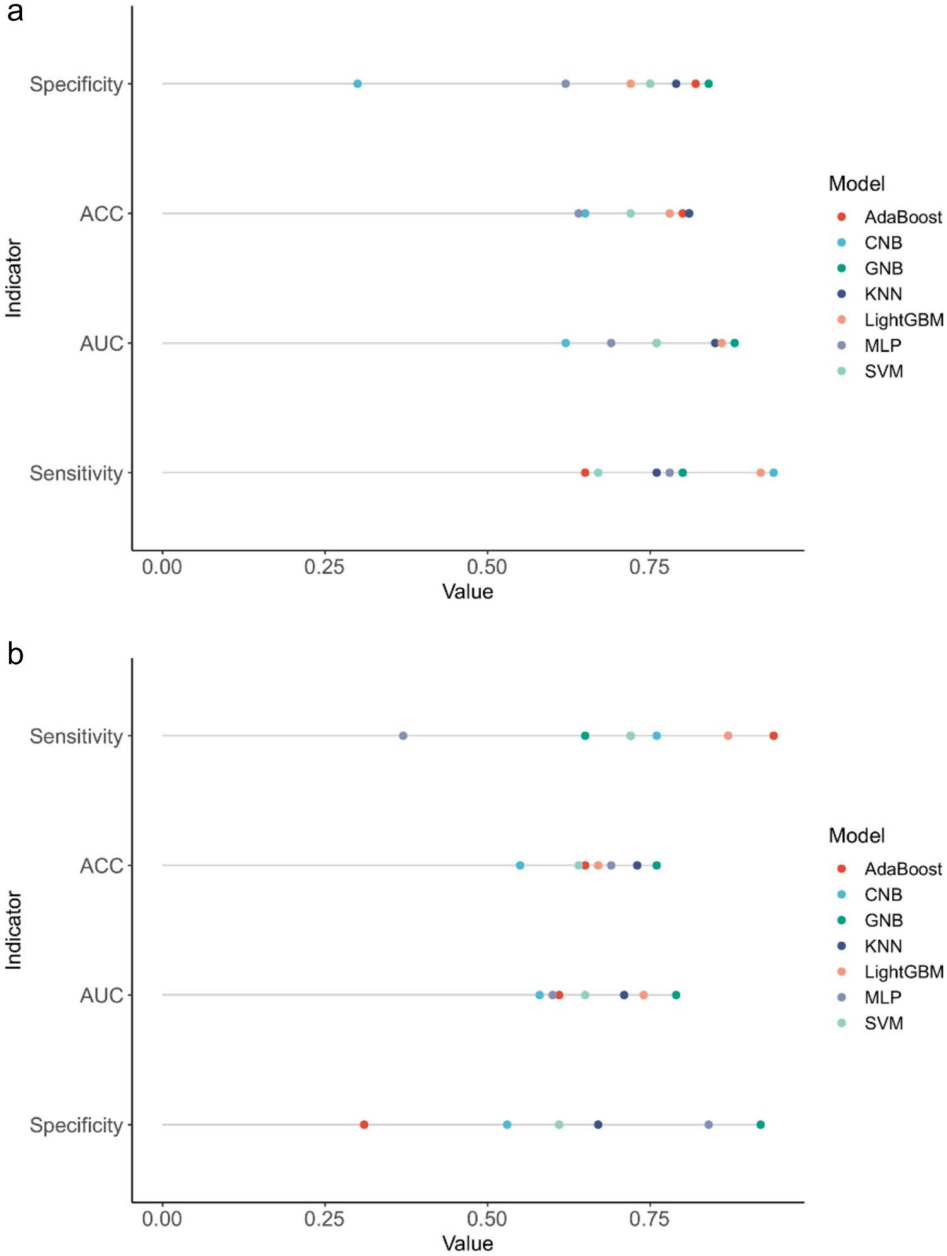
Comparisons of parameters assessing machine learning-based model performance. **a** Parameters of training models. **b** Parameters of validation models. ACC, accuracy; AUC, area under the receiver operating characteristic curve (ROC); CNB, Complement Naïve Bayes; GNB, Gaussian Naïve Bayes; MLP, multi-layer perceptron neural network; SVM, support vector machine; KNN, k-nearest neighbors

**Fig. 5:**
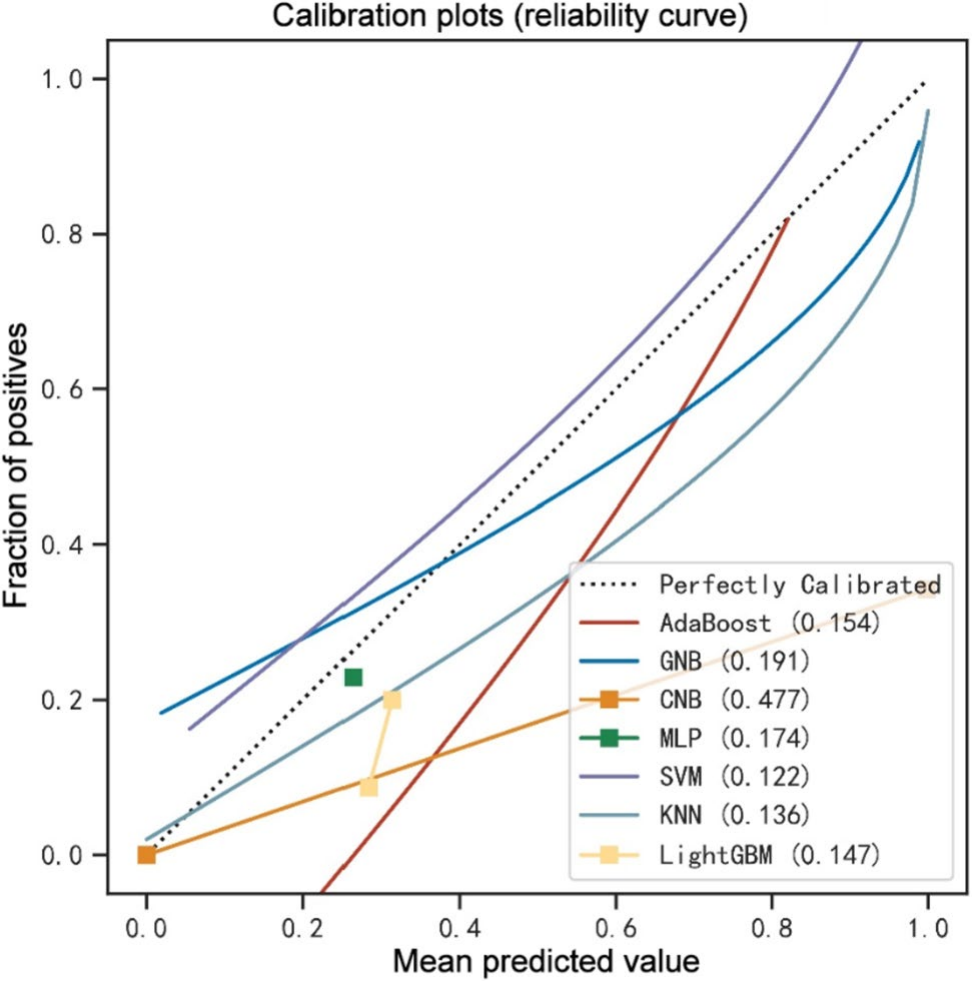
The calibration plots in the models. GNB, Gaussian Naïve Bayes; CNB, Complement Naïve Bayes; MLP, multi-layer perceptron neural network; SVM, support vector machine; KNN, k-nearest neighbors

The Boruta method was employed to identify the key variables associated with CRRT in CABG patients. Ultimately, 17 out of 39 clinical parameters remained significantly associated with CRRT, and these results are presented in Fig. 2. When assessing machine learning models for predicting CRRT, the GNB model showed the highest AUC values in both the training set (0.856, 95% CI: 0.805–0.954, Fig. 3a) and validation set (0.817, 95% CI: 0.630–0.958, Fig. 3b). Furthermore, the GNB model exhibited the highest ACC and specificity in the two data sets (Fig. 4a, b). The calibration plot was generated to evaluate the difference between predicted outcomes and actual clinical outcomes. When predicting CRRT risk, the GNB model displayed excellent calibration performance (Fig. 5). Therefore, the GNB model was recognized as the final predictive model.

### Model explanation and application

**Fig. 6:**
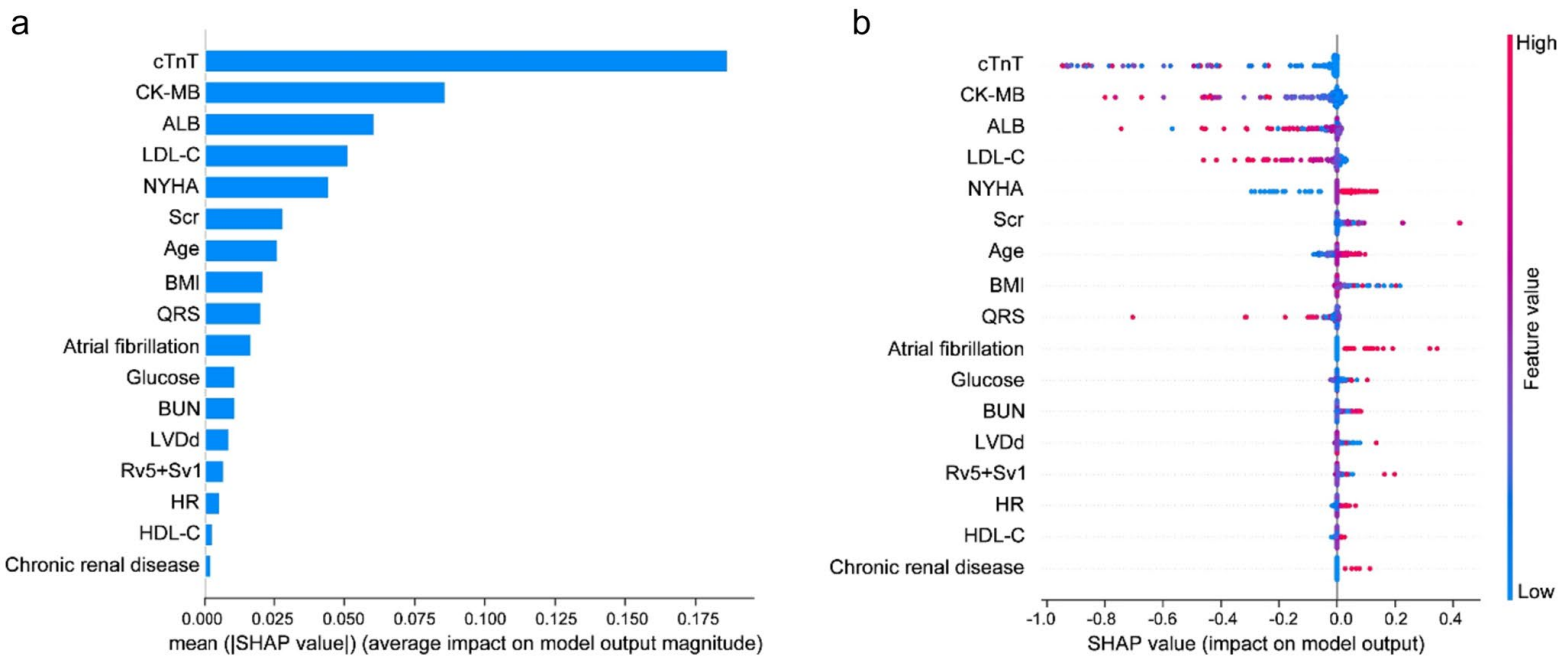
The SHAP summary plot for the clinical features contributing to the GNB model. **a** SHAP feature importance measured as the mean absolute Shapley values. This matrix plot depicts the importance of each covariate in development of the final predictive model. **b** The attributes of the features in the model. The position on the *y*-axis is determined by the feature and on the *x*-axis by the Shapley value. SHAP, SHapley Additive explanation; GNB, Gaussian Naïve Bayes

**Fig. 7:**
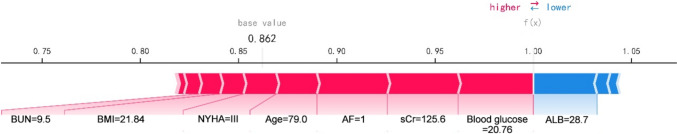
The SHAP force plot for explaining individual prediction results in the validation cohort. SHAP, SHapley Additive explanation

We next calculated the feature importance using the SHAP value for the GNB model, which had the greatest discriminatory capacity in the validation cohort. Figure 6a exhibits 17 clinical features according to the average absolute SHAP value. Figure 6b provides an overview of the impact (the positive or negative aspects) of factors on the GNB model. To further explore the contribution of the features on a certain individual patient and clinical application for the GNB model, we randomly selected one patient from the validation cohort to exhibit a visual interpretation (Fig. 7). The developed model predicted the probability of CRRT in this patient to be 86.2%. The result shows that fast blood glucose of 20.76 mmol/L, sCr of 125.6 μmol/L, atrial fibrillation, age of 79 years old, and NYHA of III classification were the top five contributors to this prediction.

## Discussion

Critically ill patients undergoing cardiac surgery frequently present with a complex clinical scenario, posing challenges for clinicians in predicting outcomes. Machine learning algorithms have proven to be valuable tools in overcoming this difficulty and are often employed to develop models for clinical studies [10]. Machine learning has emerged as a powerful tool for distinguishing and predicting prognoses in patients undergoing CABG surgery. However, these studies primarily focused on identifying risk factors associated with postoperative complications, mortality, and prolonged LOS [11–13]. Given significant impact of postoperative CRRT on hospital mortality, it was imperative to develop machine learning-based risk prediction model specifically for CRRT after cardiac surgery. Consequently, conducting an effective predictive model using preoperative clinical data is important for clinicians to provide valuable guidelines regarding appropriate interventions and rational allocation of medical resources.

This study involved the development and validation of seven machine learning models based on 17 clinical variables collected in the first 24 h of hospital admission. Among these models, the GNB model demonstrated superior predictive ability for CRRT, primarily due to its highest AUC and better calibration in this study. To achieve the best predictive performance and interpretability, the SHAP was employed in the GNB model. Feature importance analysis revealed that cTnT, CK-MB, ALB, low-density lipoprotein cholesterol (LDL-C), NYHA, sCr, and age were the top 7 features of the GNB model, with significant impact on predicting postoperative CRRT. In addition, the SHAP force analysis enables clinicians to comprehend why specific recommendations are made by the model for high-risk decisions. Collectively, these findings enhance our understanding of decision-making process underlying predictive models for users.

In this study, we have identified crucial features of postoperative CRRT. The biomarkers cTnT and CK-MB, indicating myocardial injury, could to a certain extent reflect cardiac deterioration. Preoperative elevations in cardiac biomarkers may increase the risk of postoperative complications, such as AKI [14, 15]. This could be attributed to the close relationship between cardiac and renal function, referred to as cardio-renal syndrome [15]. In our study, cTnT and CK-MB accounted for the highest weight in the GNB predictive model, which suggested that they were the most critical predictors for postoperative CRRT following CABG surgery. NYHA classification is another heart-related indicator for assessing impaired cardiac function. Several studies have explored the role of NYHA classification in predicting postoperative AKI or need for renal replacement therapy, with NYHA III/VI being an independent risk factor [16–18]. Consistently, our study showed that patients requiring CRRT had a significantly higher proportion of NYHA III/VI compared to those without CRRT (69.445% vs. 39.830%). NYHA was identified as an important risk factor of CRRT.

The crucial role of serum ALB in maintaining intravascular volume, partially by facilitating vascular integrity [19], is widely acknowledged. Consequently, reduced levels of serum ALB may result in tissue edema and decrease the circulating volume by extravasation. Previous study has demonstrated an association between preoperative low ALB and short-term or long-term prognosis in patients with cardiac surgery [20]. This suggests that reduced ALB levels can serve as prognostic indicators following cardiac surgery. In our study, we observed significantly lower ALB levels in the CRRT group compared to the non-CRRT group, albeit with a slight difference. It is worth considering that even a mild reduction in ALB levels may affect postoperative CRRT at below-normal levels. The studies have explored the relationship between preoperative renal function and postoperative CRRT, with increased sCr levels identified as the risk factor for CRRT [21–23]. Our study corroborates this finding, suggesting that patients with impaired renal function have worse tolerant ability to surgery, which increases their need for CRRT. Age is a force majeure risk factor, with older patients being less tolerant to trauma, stress, and cardiac surgery. Some clinical studies have demonstrated age as CRRT-associated risk factor after surgery in patients [21, 22]. Consistent with these findings, our study confirmed age as a predictor for postoperative CRRT after CABG surgery. Furthermore, our study also found a strong association between LDL-C and postoperative CRRT, although there was little difference in LDL-C levels between two group patients. Given that all patients had coronary artery disease, part of them may have been treated with lipid-lowering medications, such as statins. Indeed, our results showed that the rate of statin use among the two groups had no difference (69.444% vs. 77.966%). Accordingly, we hypothesis that serum LDL-C levels may be affected by different doses of statins and individual responsiveness. Despite no great difference in LDL-C levels, this indicator ought to be highly noticed in CABG patients.

Some previous researches have incorporated preoperative, intraoperative, and postoperative indicators of patients undergoing CABG to develop predictive models for clinical outcomes. It is important to acknowledge that including all variables in the model can enhance risk identification. However, it should be clarified that modeling using patients’ preoperative parameters (within 24 h after admission) offers a priori advantages in early risk variable identification and clinical guidance. Although the machine learning models demonstrated favorable predictive performance in this study, it was important to acknowledge limitations of this study. First, the retrospective nature of the study could have introduced some bias into the results. Second, external data were not used for model validation, potentially impacting the generalizability of the models. Additionally, the usage of angiotensin receptor blockers (ARBs) is reported as an important risk factor for the development of AKI. However, the data on ARBs were lacking in our study, which may affect the development of models and risk identification to some extent. We aim to address and improve these issues in future studies.

## Conclusion

Machine learning algorithm was utilized to develop a predictive model for CRRT after CABG surgery in the ICU patients, and the GNB model exhibited an excellent predictive performance and identified risk variables associated with CRRT. This study provides theoretical guidance for surgical physicians and enables the optimization of perioperative managements for patients.

## Abbreviations

ALB: Albumin
AKI: Acute kidney injury
AMI: Acute myocardial infarction
AUC: Area under the receiver operating characteristic curve
BNP: B-type natriuretic peptide
BUN: Blood urea nitrogen
CABG: Coronary artery bypass grafting
CK-MB: Creatine kinase isoenzyme
CNB: Complement Naïve Bayes
CV: Cross-validation
CRRT: Continuous renal replacement therapy
cTnT: Cardiac troponin T
GNB: Gaussian Naïve Bayes
HDL-C: High-density lipoprotein cholesterol
HR: Heart rate
IABP: Intra-aortic balloon pump
ICU: Intensive care unit
KNN: K-nearest neighbors
LDL-C: Low-density lipoprotein cholesterol
LOS: Length of hospital stay
LVEF: Left ventricular ejection fraction
LVFS: Left ventricular fraction shortening
MLP: Multi-layer perceptron neural network
SVM: Support vector machine
sCr: Serum creatinine
SHAP: SHapley Additive exPlanations
TG: Triglyceride
TC: Total cholesterol
